# Exploring patient and professional perspectives on implementing pharmacogenomic testing in the UK primary care setting and estimating the cost-effectiveness: a mixed-methods study protocol

**DOI:** 10.1136/bmjopen-2025-104311

**Published:** 2025-07-22

**Authors:** Sadaf Qureshi, Asam Latif, Dyfrig A Hughes, Stephen Timmons, Anthony Avery

**Affiliations:** 1 Centre for Academic Primary Care, School of Medicine, University of Nottingham, Nottingham, UK; 2 School of Health Sciences, University of Nottingham, Nottingham, UK; 3 Centre for Health Economics and Medicines Evaluation, Bangor University, Bangor, UK; 4 Centre for Health Innovation, Leadership and Learning, Business School, University of Nottingham, Nottingham, UK

**Keywords:** Primary Care, GENETICS, Genomic Medicine, HEALTH ECONOMICS, Protocols & guidelines

## Abstract

**Abstract:**

**Introduction:**

Pharmacogenomic testing could potentially reduce the number of adverse drug reactions and improve treatment outcomes through tailoring treatment to an individual’s genetic makeup. Despite its benefits and the ambitions to integrate into routine care, the implementation of pharmacogenomic testing in primary care settings remains limited. This study aims to qualitatively explore the views of healthcare professionals (HCPs) and patients on implementing pharmacogenomic testing in the UK National Health Service (NHS) primary care setting and to estimate the cost-effectiveness of service-delivery implementation by comparing different HCPs’ models of care.

**Method:**

This study consists of three workstreams (WS). WS1 is semi-structured interviews with General Practitioners, pharmacists, nurses and patients (24 participants) to explore implementation issues, including the perceived barriers and facilitators to delivering a pharmacogenomic service. WS2 consists of focus groups (between 24–36 participants) with genomic experts to develop practical pharmacogenomic-guided clinical pathways for primary care. WS3 will estimate the cost-effectiveness of implementing pharmacogenomic testing when led by different HCPs incorporating parameters from the literature, expert opinions, as well as data from WS1 and WS2.

**Analysis:**

Thematic analysis will be used to analyse the qualitative data from WS1 and WS2, mapping findings onto the Consolidated Framework for Implementation Research domains, which will also be used as the theoretical framework. WS3 will be a decision-analytic model developed in Microsoft Excel to compare the cost-effectiveness of pharmacist-led, GP-led, nurse-led or multidisciplinary pathways.

**Ethics and dissemination:**

This study has been approved by the NHS Health Research Authority and Health and Care Research Wales (24/PR/1088). Findings will be disseminated through peer-reviewed publications, conference presentations and engagement with NHS policymakers and Genomics England.

**STRENGTHS AND LIMITATIONS OF THIS STUDY**

- This is a mixed-methods study triangulating qualitative findings with the literature to inform an economic model.
- This study is ‘theory-driven’, informed using a theoretical framework (Consolidated Framework for Implementation Research).
- The data collected and the resulting economic model may only be applicable to the UK National Health Service primary care setting.
- Participation in the qualitative work (interviews and focus groups) was limited to self-selected individuals and those who spoke English.
- The data captured and the model are cross-sectional, and there is no objective assessment of their applicability or evaluation in the long term.

## Introduction

 Pharmacogenomic (or pharmacogenetic) testing aims to optimise prescribing to improve treatment outcomes by maximising the benefit of treatment and/or minimising the risk of harm based on genomic information.^1^ The National Health Service (NHS) in the UK has the ambition to be the first healthcare system to mainstream the use of pharmacogenomics, including in the primary care setting.^2^

Pharmacogenomic testing has the potential to identify individuals with a genetic predisposition to adverse drug reactions (ADRs) or non-response and hence facilitate safer and more effective prescribing. Pharmacogenomic testing will be particularly important for primary care, as 90% of healthcare is delivered through this setting.^3^ A systematic review^4^ in 2021 estimated the incidence of ADRs in primary care at approximately 8% with higher rates seen in very young children, older adults and females. The estimated cost to the NHS for these ADRs is approximately £2 billion per year.^5^ Pharmacogenomic testing offers the potential to mitigate the occurrence of ADRs by predicting an individual’s response to a specific medicine.^6^ This enables the clinician to alter the drug dosage or substitute with a complementary medicine, thereby reducing the incidence of ADRs,^6^ improving efficacy, minimising clinic visits and improving adherence.^7 8^ The focus of most current pharmacogenomic tests has been in the context of reducing the likelihood of ADRs.^9^ However, pharmacogenomic information can also help improve drug effectiveness. For example, some drugs need to be activated by enzymes in the body, and genetic differences can affect how well this happens, such as CYP2D6 with codeine or CYP2C19 with clopidogrel; or genetic changes in drug targets can influence how well a drug works, such as VKORC1 (vitamin K epoxide reductase complex subunit 1) with warfarin or CFTR (Cystic Fibrosis Transmembrane Conductance Regulator) with ivacaftor. Collectively, these factors highlight the growing relevance of pharmacogenomic testing in primary care.

The clinical utility of certain single drug-gene pharmacogenomic tests relevant for primary care has been demonstrated in clinical trials, for example, warfarin for atrial fibrillation^10^ and pharmacogenomic testing in mental health disorders.^11^ Furthermore, the clinical utility of a multigene pharmacogenomic panel was established by the PREPARE study,^6^ which demonstrated that pharmacogenomic testing can reduce ADRs by 30% for patients prescribed certain medicines. This reduction could potentially lead to fewer repeat visits to primary care and thereby ease pressures on general practice by freeing up time and resources. Such benefits are particularly relevant given the current strain on general practitioner (GP) appointments in primary care, the ageing population and the increasing prevalence of patients with multiple long-term conditions.^12^ These demographic and clinical trends often result in polypharmacy and consequently increased risk of ADRs.^13^ Economic evidence is similarly encouraging; a recent systematic review^14^ reported that 71% of 108 studies found pharmacogenomic testing for drugs with existing guidelines to be either cost-effective or cost-saving.

For the purpose of this study, primary care in the UK consists of general practices and community pharmacies. Traditionally, the general practice model included GPs and nurses. However, over the past decade, general practice has seen an expansion of different healthcare professionals (HCPs) working collaboratively. These HCPs include pharmacists, pharmacy technicians, healthcare assistants, physician assistants, physiotherapists and paramedics, with many of these HCPs having prescribing rights. Therefore, understanding the roles of these diverse HCPs in primary care is crucial for the successful implementation of pharmacogenomic testing, as their involvement in patient care and prescribing decisions will shape how pharmacogenomic information is integrated into routine practice.

Although the clinical and cost-effectiveness of a number of pharmacogenomic tests has been demonstrated through various systematic reviews,^15 16^ implementation issues remain. To ensure an efficient and uniform uptake of pharmacogenomic testing across primary care, it is essential to identify the barriers and facilitators to implementation and determine which HCPs should coordinate its delivery. Several international studies have identified these barriers and facilitators;^17^ however, a UK NHS study involving the range of primary care HCPs and patients about these barriers and facilitators is lacking.^18^ Moreover, given the current financial constraints facing the NHS, it is important to understand the costs and value of implementing pharmacogenomic testing in primary care.

### Aims and objectives

This study aims to investigate the perceived barriers and facilitators to the implementation of pharmacogenomic testing in the UK primary care setting. Furthermore, it seeks to assess the value of implementing such testing when led by GPs, pharmacists or nurses.

This study is divided into three workstreams (WS) with the following objectives:

- **WS1**—To explore the views of UK patients and HCPs, which includes GPs, practice pharmacists, community pharmacists and practice nurses, regarding the perceived barriers and facilitators for the implementation of pharmacogenomics into routine primary care practice, using the Consolidated Framework for Implementation Research (CFIR).
- **WS2**—To build on the findings of WS1 to develop pharmacogenomic-guided clinical pathway(s)/workflow(s). HCPs with expertise in genomics and patients will consider the operational changes for implementation, as well as the division of roles and responsibilities through relevant stakeholder engagement.
- **WS3**—To estimate the cost-effectiveness of implementing pharmacogenomic testing in primary care led by GPs, pharmacists or nurses.

## Methods and analysis

This study began in September 2024 and is due to continue until September 2026. The study will employ a mixed methods approach involving three WS. These are outlined below and then described in more detail.

- **WS1** will involve semi-structured interviews with HCPs, including GPs, pharmacists, nurses and patients.
- **WS2** will involve focus groups, both conducted with HCPs and patients. In conjunction with WS2.
- **WS3** will develop several models of pharmacogenomic-guided clinical pathways to estimate the cost-effectiveness of implementation by different HCPs. These model pathways will be presented to participants in the focus groups of WS2.

### Qualitative methods

#### WS1: semi-structured interviews with HCPs and patients

##### Recruitment

This WS will focus on the views of GPs, pharmacists, practice nurses and patients. Participants will be recruited using a purposive sampling method, which is a non-probability approach, to ensure a diverse representation across various demographics. Recruitment for the study will include contact and promotion through the main researchers’ professional networks in primary care (including contacts through previous employment and membership of genomics networks), supervisor contacts, circulation of study flyers via high street pharmacies, GP surgeries, social media, through regional Research Design Networks and snowball sampling.

Eligibility criteria include those HCPs currently working in primary care and patients >18 years of age and receiving medicines from their GP. WS1 will recruit twenty-four participants, including six GPs, three practice-based pharmacists, three community pharmacists, six nurse prescribers and six patients. The study aims to include participants from a wide range of demographics, including age, years since qualifying, gender and ethnicity, to ensure a wide range of opinions are included. Twenty-four participants were considered a sufficient sample size due to the specific aims of the study,^19^ other qualitative studies have used a similar sample size,^17^ and this participant size is considered sufficient to reach data saturation.^20 21^ A systematic review by Hennink and Kaiser^22^ suggests that most qualitative studies can reach saturation at relatively small sample sizes. Furthermore, this sample size was chosen based on the pragmatic decision of available resources and timelines.

##### Data collection

Qualitative data will be collected through semi-structured interviews, expected to last between 45–60 mins. Interviews will be offered either online via MS Teams or face-to-face, depending on the participants’ preference. Interviews will be recorded on an encrypted audio device. Written consent to participate will be obtained prior to the interview. Recordings will be stored on a secure server. Interview scripts will be transcribed, anonymised and coded. Interview guides for patients and HCPs will be developed separately and reviewed by the supervisory team (see onlinesupplemental files 1  2). Questions will explore the perceived barriers and facilitators for implementing pharmacogenomic testing in primary care, focusing on the unique challenges of how different HCPs could integrate pharmacogenomics into their roles and responsibilities to optimise patient care. Demographic information, including age, sex, ethnicity and education level, will be collected from each participant before the interview.

### WS2: focus groups with genomic experts, stakeholders and patients

The focus of WS2 is to present a series of plausible HCP-led pharmacogenomic-guided clinical pathways to a panel of experts and patients to evaluate their practicality, feasibility and cost for use in primary care.

#### Recruitment

Four focus groups will be set up, each with 6-9 participants. These participants will include GPs, pharmacists and nurses with some expertise in genomics, commissioners, medical directors, chief pharmacists, laboratory scientists, specialists from secondary care and patients. Each focus group will be heterogeneous with diverse participants, to ensure different opinions are explored. A similar recruitment strategy to WS1 will be employed for WS2, but with an emphasis on recruiting HCPs with some genomics expertise. The sample size of the focus groups is considered sufficient to generate sufficient data to meet the aim of this WS. Guest *et al*^23^ have suggested that 80% of all themes are discovered within 2–3 focus groups.

#### Data collection

Each focus group will last a minimum of 90 mins and up to a maximum of 120 mins. The focus groups will be offered either online via MS Teams or face-to-face, depending on the participants’ preference and convenience. Interviews will be recorded on an encrypted audio device and supplemented with handwritten notes. Participants will be given an identifying code to enable anonymisation (eg, GP1, P1, N1, etc). The main barriers and facilitators defined in WS1 will be ordered into emerging themes and used to further define the needed actions, roles and responsibilities of the primary care team. A script including an introduction to pharmacogenomics, case scenarios and presentation of a model (see onlinesupplemental files 3  4) will be developed and participants will be asked to discuss the themes in the focus group. All data will be anonymised. Results from the focus group and semi-structured interviews will be used to develop proposed pharmacogenomic-guided clinical pathways for use in primary care.

### Qualitative data analysis for WS1 and WS2

Data obtained from the semi-structured interviews and focus groups will be transcribed verbatim and checked by the main researcher. NVivo 15 will be used to organise the data, facilitate coding and help with the analytical process. Data analysis will start during the early stages of data collection and proceed iteratively so that emergent findings are incorporated into subsequent data collection.

All transcripts will involve initial coding through an inductive approach. This will include initial reading and re-reading of the anonymised transcribed data by the main researcher to identify common codes and categories and to develop a coding frame. Codes will then be grouped into emerging themes using Braun and Clarke^20^ and King *et al*^21^ guidance on conducting thematic analysis. These codes and themes will be reviewed by the supervisory team through regular monthly meetings.

The themes will then be mapped to the CFIR to assess the emerging barriers and facilitators of implementation. The CFIR is a conceptual framework that was developed to integrate various implementation science theories into a single, comprehensive model. The CFIR is composed of five domains, including:

1. Intervention characteristics—features of pharmacogenomic testing.
2. Inner setting—features of primary care/general practice in the UK.
3. Outer setting—NHS.
4. The individuals—GPs, pharmacists, nurses and patients.
5. The process—strategies to influence the implementation of pharmacogenomics.

There are multiple constructs under each domain providing a systematic approach to identifying factors that influence the implementation of an intervention. The framework is an ideal tool to study a complex intervention such as pharmacogenomic testing, which will involve multiple stakeholders, workflows and systems. Compared with other theoretical frameworks, it is well suited for exploring roles, responsibilities and organisational changes.^24^

#### WS3: Decision-analytical modelling

The objective of WS3 is to estimate the cost-effectiveness of potential pharmacogenomic pathways in primary care. With the recent expansion of the primary care team, an increasing proportion of prescribing is undertaken by non-medical prescribers. However, there is uncertainty regarding the cost-effectiveness of different models of care for delivering pharmacogenomics in primary care.

**Figure 1:**
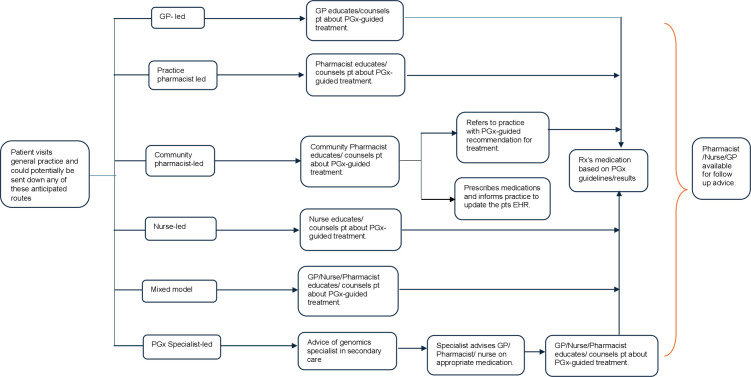
Displays some exemplar pharmacogenomic-guided clinical pathways. GP, general practitioner.

Background work for WS3 will commence prior to WS2, with the purpose of presenting a model to the focus group for discussion. Data generated from WS1 will be used to populate the model. For the focus groups in WS2, this initial model will be presented for discussion and then amended as suggested by the focus groups. The initial phase will involve systematically mapping the steps to incorporate pharmacogenomic testing into the primary care workflow. Figure 1 displays some exemplar pharmacogenomic-guided clinical pathways. These pathways are by no means exhaustive, and the focus groups will be encouraged to amend, adapt or propose new pathways. A consensus on practical pathways will be sought through engagement and agreement with the focus group participants.

A review of the literature will be conducted to identify the proposed process steps, such as pretest counselling, ordering the pharmacogenomic test and actions based on the test results. Once the process steps are mapped out, a matrix will be developed to categorise various pharmacogenomic testing models based on the HCP leading the process (eg, GP-led, pharmacist-led, nurse-led).

A decision-analytic model will be developed in Microsoft Excel to compare the cost-effectiveness of the pharmacist-led, GP-led, nurse-led or multidisciplinary approaches. This model will align with the National Institute for Health and Care Excellence (NICE) technology evaluation methods.^25^

##### Population of interest

HCPs including GPs, practice pharmacists, community pharmacists and nurses who can conduct and interpret pharmacogenomic testing in the UK primary care setting (general practice and community pharmacies).

##### Intervention

Pharmacogenomic test-guided prescribing conducted and interpreted by a GP, pharmacist, nurse or a combination of primary care professionals.

##### Comparator

Standard prescribing practices in primary care, where prescribing is undertaken by a GP without pharmacogenomic guidance.

##### Perspective on costs

This will be undertaken from the NHS and Personal Social Services perspective.

##### Time horizon

This will be the duration over which health outcomes and costs are calculated. The model will use a lifetime time horizon, which will reflect all important differences in costs or outcomes between pharmacogenomic-guided prescribing and standard prescribing in primary care.

##### Outcomes

Quality-adjusted life years (QALYs). QALYs are a measure of the state of health of a person or group in which the benefits, in terms of length of life, are adjusted to reflect the quality of life.

##### Costs

Only direct medical and personal social service costs will be included in the model. Examples of costs to be included are primary care clinicians’ time to consult a patient, laboratory testing costs, test costs, drug costs and alternative drug treatment costs.

### Analytical strategy

A decision analytic model will be constructed for a cohort of typical general practice hosting GPs and non-medical prescribers. The cohort will be modelled over a lifetime. The model will have a structure which reflects a typical patient journey through a GP practice. The model seeks to address the issue of technical efficiency—what is the most efficient model of service delivery, as opposed to allocative efficiency (whether testing is undertaken) and as such, the comparators will be alternative HCPs delivering the service. The intervention will be the use of a pharmacogenomic test to guide prescribing by a prescriber (GP or non-medical). A systematic literature review including existing economic evaluations, randomised controlled trials, observational studies and meta-analyses will be undertaken to inform the inputs of the model. Where there is a gap in the literature, the expert focus group in WS2 will be engaged to inform the inputs in the model. All costs and outcomes will be discounted at 3.5% per annum (as recommended by NICE).

The cost-effectiveness of the new way of working will be calculated using the incremental cost-effectiveness ratio, which is the difference in costs between the intervention and standard care divided by the difference in effect between the intervention and standard care. This will be compared with NICE thresholds for cost-effectiveness.

### Sensitivity analyses

Sensitivity analyses^26^ will be performed. Deterministic sensitivity analyses will be used to determine variation for a set of parameters. This will involve varying the model input values and recording the impact of these changes on the model outputs. Probabilistic sensitivity analyses will be used to characterise the joint distribution of uncertainty in costs and QALYs. The outputs will be reported in terms of the probability of each pathway being cost-effective.

## Discussion

This study will provide valuable evidence into the perspectives of both HCPs and patients on the use of pharmacogenomic testing in the primary care setting in the NHS, an area where current evidence remains limited. It will also explore the implementation of pharmacogenomic testing through clinically plausible, HCP-led pathways to assess their practicality, feasibility and cost for use in primary care.

In addition to capturing stakeholders’ views, the study will also generate economic evidence concerning the integration of pharmacogenomic testing into routine practice. This cost-effectiveness analysis will be particularly informative for decision-makers, offering them essential evidence to guide future implementation strategies and resource allocation.

### Study limitations

One of the main limitations of this study is that the sampling, data collection, coding, analysis and interpretation will be undertaken by the main researcher, but guided and supervised by the research team. The main researcher, as a practising pharmacist and woman of colour, brings her own inherent biases into the way the research is conducted. However, with the aid of a strong supervisory team, these biases may be limited. In terms of representation of participants, efforts will be made to include a wide cohort, representing different voices across the HCPs, ages, genders and ethnicities. However, participants in this study will be those that are interested in the research topic and may not be reflective of the general population. This study focuses on the primary care setting in the NHS, a health service which is publicly funded and therefore its applicability to other healthcare settings will be limited.

### Ethics and dissemination

The NHS Health Research Authority and Health and Care Research Wales (24/PR/1088) have approved this study protocol. All participants will receive written study information about the purpose of the study, background information about pharmacogenomic testing and the consent form. Results from this study will be presented at scientific meetings. Publications will be through a written doctoral thesis and journal publications, and conferences. Findings will be disseminated to NHS England/Genomics England. Participants will not be identified in any publication. This study is part of an educational qualification for a PhD. A summary of the study will be provided to the funder.

### Patient and public involvement

This study was presented to a patient and public panel. The panel was supportive of the study, with the consensus that pharmacogenomics could help with particularly difficult-to-treat patients, especially those who had polypharmacy. Further, I have a PPI member who has and will continue to be involved in the study.

## Supplementary material

10.1136/bmjopen-2025-104311 online supplemental file 1

10.1136/bmjopen-2025-104311 online supplemental file 2

10.1136/bmjopen-2025-104311 online supplemental file 3

10.1136/bmjopen-2025-104311 online supplemental file 4
