# Supplementary material for: Exploring patient and professional perspectives on implementing pharmacogenomic testing in the UK primary care setting and estimating the cost-effectiveness: a mixed-methods study protocol
Source: BMJ Open. 2025 Jul 22;15(7):e104311. doi: 10.1136/bmjopen-2025-104311 (PMC12306336; doi:10.1136/bmjopen-2025-104311)
Supplement: online supplemental file 1 [file bmjopen-15-7-s001.docx]

**Topic guide**

**Workstream1: Semi structured interview with Healthcare Professionals (HCP)**

**Objective: views of pts and HCP on perceived barriers and facilitators for implementation of PGx into primary care.**

- *Introduce self.*
- *Explain the purpose of the interview.*

Thank you for agreeing to participate in this study. As you are aware we are interested in learning more about your thoughts, insights, and perceptions about the use of pharmacogenomic testing to guide prescribing in primary care. There are no right or wrong answers.

*The interview will take approximately 45-60 mins.*

**Checks before proceeding.**

- Check that the participant has received the information sheet and signed the consent form.
- Ask for consent to record the interview. Explain that recordings will only be accessed by the research team and will be stored securely.
- Confirm that any quotes used will not be linked to any individual. No individuals will be identified in the reporting.
- Is the participant willing to take part in the interview?

**Thinking about PGx testing**

- What do you understand about the term PGx?

**For HCPs**

- Have you ever heard of PGx testing before enrolling in this study?
- Do you think this is a term that would be easily understood by GPs/Pcists?
- What are your current experiences, if any with pharmacogenomic testing in your practice?

**Source (innovation domain)**

- Do you feel you have had the right amount of training/information/awareness to enable you to confidently utilise PGx testing in your routine work? Why or why not?
  - Would you have any training or educational needs?

**Evidence** **(innovation domain)**

- What do you see as the potential benefits of pharmacogenomic (PGx) testing?
- What do you think could be the challenges in the use of PGx test results/data? (e..g interpretation, understanding, clinical utility etc)
  - How could we overcome those challenges?
- Are there any practical / logistical barriers to implementing PGx testing in primary care – either for the patient or for you, the HCP?
- Are there any particular patient populations or conditions you feel would particularly benefit from PGx testing? E.g. people with polypharmacy

**Complexity (innovation domain)**

- Would the extra time needed to discuss/explain PGx test results with patients affect your decision to use it?
  - Would you consider delegating this to another healthcare professional, like a pharmacist or nurse?
- How likely are you to consider using PGx test results to guide medication choice for your patients? Why or why or not
- Do you have any particular concerns about using PGx testing?
- Are there any particular resources that would help you to use PGx testing? In reality how easy would these be to introduce? (e.g CDS/EHR)

**Miscellaneous**

- If PGx involves various members of the primary care team, what role do you think a GP or Pcist or nurse could play?
- Do you think Community Pharmacist or Practice based pharmacists should be involved in PGx testing?
  - Should PGx test results be shared with all HCPs? (ie Community Pharmacy)
- How should PGx information be stored?
- How should we raise awareness in the general public about PGx testing? i.e. national campaign?
- Is there anything else related to PGx testing that we haven’t discussed already that you feel is important?
