# Supplementary material for: Exploring patient and professional perspectives on implementing pharmacogenomic testing in the UK primary care setting and estimating the cost-effectiveness: a mixed-methods study protocol
Source: BMJ Open. 2025 Jul 22;15(7):e104311. doi: 10.1136/bmjopen-2025-104311 (PMC12306336; doi:10.1136/bmjopen-2025-104311)
Supplement: online supplemental file 2 [file bmjopen-15-7-s002.docx]

**Topic guide**

**Workstream1: semi structured interview with patients**

**Objective: views of pts and HCP on perceived barriers and facilitators for implementation of PGx into primary care.**

- *Introduce self.*
- *Explain the purpose of the interview.*

Thank you for agreeing to participate in this study. As you are aware we are interested in learning more about your thoughts, insights, and perceptions about the use of pharmacogenomic testing to guide prescribing in primary care. There are no right or wrong answers.

*The interview will take approximately 45-60 mins.*

**Checks before proceeding.**

- Check that the participant has received the information sheet and signed the consent form.
- Ask for consent to record the interview. Explain that recordings will only be accessed by the research team and will be stored securely.
- Confirm that any quotes used will not be linked to any individual. No individuals will be identified in the reporting.
- Is the participant willing to take part in the interview?

Before we talk about pharmacogenomic testing, I have some general questions I would like to ask:

- How do you feel about taking prescribed medicines in general?
- What concerns or questions do you have when starting a new medicine?

**General awareness and initial perceptions**

- Have you ever heard of PGx testing before? If so, what do you know about it?

**Personal relevance and benefits**

- What would make you want to undergo a PGx test as a patient?
- What concerns would you have when getting a PGx test?

**Privacy, trust and ethical concerns**

- Do you have any concerns about sharing your PGx/genetic information with healthcare professionals?
- How should personal pharmacogenetic information be stored?

**Practical Considerations**

- What concerns, if any, do you have about the time it might take to complete the PGx testing process? (e.g. appointments, sample collection, counselling etc.)
- How would you feel if the test results suggested changing a medication you’re already taking?
- Would the time or travel for additional appointments affect your decision to have this testing?
- How helpful would it be to have a follow-up appointment specifically to go over your PGx test results and the next steps?

**Decision-making and trust in healthcare providers**

- Would you trust your healthcare provider to recommend this type of PGx/genetic testing for you? Why or why not?
- Do you think knowing your genetic information might make you feel more worried or more empowered about your health?

**Benefits**

- What do you think are the benefits of introducing PGx in primary care?
- Would you be willing to try PGx testing if it could be done at a pharmacy/High Street chemist or any other location?
- Which HCP would you like to see delivering PGx i.e. ordering, testing, relaying information?

**Awareness**

- How should we raise awareness in the general public about PGx testing? i.e. national campaign?
- Is there anything else related to PGx testing that we haven’t discussed already that you feel is important?
