# Supplementary material for: Exploring patient and professional perspectives on implementing pharmacogenomic testing in the UK primary care setting and estimating the cost-effectiveness: a mixed-methods study protocol
Source: BMJ Open. 2025 Jul 22;15(7):e104311. doi: 10.1136/bmjopen-2025-104311 (PMC12306336; doi:10.1136/bmjopen-2025-104311)
Supplement: online supplemental file 3 [file bmjopen-15-7-s003.docx]

**Topic guide – workstream 2: focus group with HCPs**

**Structure of focus group:**

- Welcome and Introductions
- Objective and Consent
- Icebreaker: Experience with genomics
- Baseline Understanding: "What does PGx mean to you?"
- Short Presentation of PGx
- Case Scenario 1: Reactive Testing
  - Presentation and discussion
- Case Scenario 2: Pre-emptive Testing
  - Presentation and discussion
- Present model
  - Role Mapping & Responsibilities Discussion
- Wrap-Up and Thanks

**Objective:** to explore how pharmacogenomic (PGx) testing could be integrated into primary care workflows, identify the most appropriate HCPs to deliver various elements of PGx and understand operational implications through stakeholder discussion of case scenarios.

- *Introduce self.*
- *Explain the purpose of the interview.*

Thank you for agreeing to participate in this study. As you are aware we are interested in learning more about your thoughts, insights, and perceptions about the use of pharmacogenomic testing to guide prescribing in primary care. There are no right or wrong answers.

*The focus group will take approximately 90-120 mins.*

**Checks before proceeding.**

- Check that the participant has received the information sheet and signed the consent form.
- Ask for consent to record the interview. Explain that recordings will only be accessed by the research team and will be stored securely.
- Confirm that any quotes used will not be linked to any individual. No individuals will be identified in the reporting.
- Is the participant willing to take part in the interview?

**Icebreaker:**

Please could you introduce yourself and share if you have any experience of using genetics/genomics in our everyday work.

To ease participants in, ask the following question:

1. What you understand by the term pharmacogenomics?

Very short power point presentation

Present the two case scenarios for discussion.

**Scenario 1: Reactive testing**

**Patient profile**

Name: Mrs Minnie Mouse

Age: early fifties

Presenting complaint: Ongoing moderate musculoskeletal pain (mainly at night), interfering with sleep and daily function

Diagnosis: osteoarthritis

Previous treatments: ibuprofen PRN

Mrs. Mouse has been using **codeine 30 mg QDS for 10 days**, prescribed by her GP for chronic osteoarthritic pain. However, she reports minimal pain relief, despite good adherence and no significant side effects.

Given her lack of response, the prescriber considers whether pharmacogenomics may be a factor. A PGx test is arranged, and results show:

**CYP2D6 genotype:** Poor metaboliser

**Pharmacogenomic Relevance**

Codeine is a prodrug that requires CYP2D6-mediated conversion to morphine for analgesic effect.

Poor metabolisers have little or no CYP2D6 enzyme activity, so they do not convert codeine effectively, leading to reduced or no analgesia.

Risks of side effects remain (e.g., sedation, constipation), without any pain control benefit.

**International guidelines Recommendation (e.g., CPIC, DPWG)**

- Avoid codeine due to lack of efficacy.
- Consider using non-CYP2D6-dependent alternatives, such as:
  - Morphine
  - Oxycodone
  - NSAIDs (e.g., topical or oral)
  - Consider avoiding tramadol.[^b^](https://pmc.ncbi.nlm.nih.gov/articles/PMC3289963/#tfn4-cpic_2d6-codeine)

**Discussion points**

1. What are your thoughts about using PGx results to guide prescribing?
2. Who should be involved in obtaining consent and explaining the PGx test?
3. What would work better, saliva or blood test?
4. Who should collect the sample?
5. Who should be involved in interpreting/explaining the results?
6. How should it be documented?
7. How do you feel generally about acting on PGx results?
8. Who would you ask for advice if unsure about prescribing? (e.g. clinical pharmacist? Specialist?)

**Scenario 2: pre-emptive testing using CYP2C19 genotype**

**Patient Profile**

Name: Mr John Doe

Age: late forties

Presenting complaint: persistent low mood, sleep disturbance, low energy and poor appetite for over 6 weeks

Diagnosis: Depression

Previous treatments: none for depression

Social: employed, no alcohol or drug use

You consider citalopram for John Doe. During the consultation the prescriber notes that John previously had a pharmacogenomic test ahead of clopidogrel prescribing. His genotype is documented as CYP2C19 *17/*17 **ultrarapid metaboliser**.

**Background**

Citalopram is metabolised the CYP2C19 gene. In ultrarapid metabolisers, increased CYP2C19 enzyme activity leads to faster metabolism of citalopram.

This increased metabolism of citalopram leads to less active compounds.

Lower plasma concentrations decrease the probability of clinical benefit

**Internation guidelines (**[CPIC](chrome-extension://efaidnbmnnnibpcajpcglclefindmkaj/https:/files.cpicpgx.org/data/guideline/publication/serotonin_reuptake_inhibitor_antidepressants/2023/37032427.pdf) / [PharmGKB](https://www.pharmgkb.org/guidelineAnnotation/PA166127638))

- Consider a clinically appropriate alternative antidepressant not predominantly metabolised by CYP2C19.
- If citalopram is clinically appropriate, and adequate efficacy is not achieved at standard maintenance dosing, consider titrating to a higher maintenance dose

Through shared decision-making with the patient, it is decided to start John on an alternative SSRI - fluoxetine which is not metabolised by CYP2C19 gene.

**Discussion points**

1. What are your thoughts about the risks of ignoring the PGx results?
2. How do you feel generally about acting on PGx results like this?
   1. In the context of this particular case how likely or willing would you be to accept the PGx results and change the medication?
3. You already have the results in your record, what is a practical way to identify these results when needed for prescribing another medication?
4. Whose responsibility would it be to ensure this was set up?
5. Who should be involved in interpreting/explaining the results?
6. Who should be involved in counselling the patients?
7. Who would you ask for advice if unsure about prescribing? (e.g. clinical pharmacist? Specialist?)
