# Supplementary material for: Exploring patient and professional perspectives on implementing pharmacogenomic testing in the UK primary care setting and estimating the cost-effectiveness: a mixed-methods study protocol
Source: BMJ Open. 2025 Jul 22;15(7):e104311. doi: 10.1136/bmjopen-2025-104311 (PMC12306336; doi:10.1136/bmjopen-2025-104311)
Supplement: online supplemental file 4 [file bmjopen-15-7-s004.docx]

**Topic guide - Workstream 2: Focus group with patients.**

Objective: to develop pharmacogenomic clinical pathways/workflow with genomic experts HCPs and patients, to consider the operational changes for implementation as well as the division of roles and responsibilities through relevant stakeholder engagement.

- *Introduce self.*
- *Explain the purpose of the interview.*

Thank you for agreeing to participate in this study. As you are aware we are interested in learning more about your thoughts, insights, and perceptions about the use of pharmacogenomic testing to guide prescribing in primary care. There are no right or wrong answers.

*The focus group will take approximately 90-120 mins.*

**Checks before proceeding.**

- Check that the participant has received the information sheet and signed the consent form.
- Ask for consent to record the interview. Explain that recordings will only be accessed by the research team and will be stored securely.
- Confirm that any quotes used will not be linked to any individual. No individuals will be identified in the reporting.
- Is the participant willing to take part in the interview?

*I will ask questions on the following areas (there are some example questions below, but these may change on an iterative basis):*

**Future implementation:**

- In the next 5 years, do you think PGx testing will be implemented in primary care?

**Infrastructure:**

- Which HCP would you want to be involved in PGx testing (this can include ordering, interpreting, delivering and counselling)?

**Actors:**

- Discussion on the healthcare professionals involved in PGx testing in primary care:
- Who requests the PGx test and when?
- Who is giving information about the test?
- Who interprets the test results?
- Who discusses the results of the test (with whom)?
- Does the patient receive other information (e.g. pamphlet/gene-profile on a card)?
- What is role of pharmacists?
- What is role of GPs?
- What is the role of nurses?
- What is role of the lab?
- Role of the patient?

**Procedure/roles and responsibilities:**

- How do you see PGx testing applied in primary care?

**Information-exchange:**

- What information is relevant?
- How should that information be provided (orally/written/online etc.)?
- When should this information be provided?
- Where would you want to go if you have further questions?

**Organisation:**

- When should the test be done?
- Where is the sample (blood/saliva) taken?
- How long would you want to wait for the results?
- What happens after the test-results?
- Who pays for the test?

**Implementation:**

• Which factors are relevant for implementation of pharmacogenetics in primary care?

• Which factors inhibit implementation of pharmacogenetics in primary care?

*I will present some suggested pharmacogenomic clinical pathways with various HCP involved at different steps and ask for comments, modifications or if not acceptable to redo the pathways together.*

This will conclude the focus group.
