# Supplementary material for: Exploring patient and professional perspectives on implementing pharmacogenomic testing in the UK primary care setting and estimating the cost-effectiveness: a mixed-methods study protocol
Source: BMJ Open. 2025 Jul 22;15(7):e104311. doi: 10.1136/bmjopen-2025-104311 (PMC12306336; doi:10.1136/bmjopen-2025-104311)
Supplement: Supplementary file 5 [file bmjopen-2025-104311.reviewer_comments.pdf]

PEER REVIEW HISTORY

BMJ Open publishes all reviews undertaken for accepted manuscripts. Reviewers are asked to complete a checklist review form and are provided with free text boxes to elaborate on their assessment. These free text comments are reproduced below.

ARTICLE DETAILS

Title (Provisional)

Exploring patient and professional perspectives on implementing pharmacogenomic testing in the UK primary care setting and estimating the cost-effectiveness: A Mixed-Methods Study Protocol

Authors

Qureshi, Sadaf; Latif, Asam; Hughes, Dyfrig A; Avery, Tony; Timmons, Stephen

---

VERSION 1 - REVIEW

**Reviewer**	**1**
**Name**	**Wright, David**
**Affiliation**	**University of Leicester, School of Healthcare**
**Date**	**09-Jun-2025**
**COI**	**None**

The authors focus the value of PGx on reduction in ADRs however they also improve therapeutic effectiveness as they inform which drugs may be more appropriate to start in a patient meaning that meaningful response is received in a more timely manner as a result. The background would be better written such that what PGx does and how it should be used is clearly stated and explained.

PGx is already delivered nationally through community pharmacies in the Nederlands and GP practices in Norway as part of their national health systems. I am unsure if community pharmacy is not included in the definition of 'primary care'. It is also available as a private service widely in primary care in Australia, Canada and USA. A GP based service is currently being trialled as an implementation study (The Progress Trial) in Manchester, UK, which includes GPs and Pharmacists. An economic model is being built by a health economist as a result,

I therefore think the background needs to be more clear as to where this project sits and what it will really add as is seems to suggest that the research will be performed on those naive to the use of PGx. It would be far more useful to perform in on those who have used it in primary care.

It is also very clear that most healthcare professionals have limited understanding of what PGx is and what it isn't. Therefore qualitative work to understand their perspectives is likely to not add a lot of value as views will be all theoretical.

I would have expected WP1 to be a review of the literature, as this would provide much better evidence and information for building an economic model of this nature that qualitative views from intervention ignorant and naive individuals.

Different drugs have different quality of evidence for PGx and different actionable rates and different companies offer different panels. The NHS is starting to coalesce its thoughts as to what drugs it would like to include, with Clopidogrel already being planned. It may be easier and cleaner to focus on one drug with known outcomes and adverse events rather than panels which have different outcomes for each drug.

The authors talk about implementation and again this may differ for different therapies. Delaying the use of some treatments in anticipation of the PGx result may be appropriate in some instances but not others. It may be better to build models based on different drugs rather than panels and use this to inform which drugs should eventually be included in panels.

Part of implementation is whether HCPs actually offer the test. The authors allude to this as being part of the problem but I am unsure if the level of implementation (reach) or quality of response to test results (dose/fidelity) will be considered in the model. PGx evidence comes from unstructured observational data and as such is of medium quality at best. Therefore, again, a good proportion of results will be incorrect for the patient, particularly if they are non-white. Again the authors do not seem to recognise these issues in their plans.

The qualitative work requires review by a qualitative researcher. Either the authors are going to treat the data from the interviews as heterogeneous and therefore the planned numbers for some healthcare professional groups seem very small, or homogeneous and therefore a justification for different numbers for each group is required i.e. why should some voices get more say than others.

The paper also needs reviewing by a health economist with expertise in modelling as I am unable to comment on this element.

VERSION 1 - AUTHOR RESPONSE

Reviewer: 1 Dr. David J Wright, University of Leicester. Comments to the Author:	
The authors focus the value of PGx on reduction in ADRs however they also improve therapeutic effectiveness as they inform which drugs may be more appropriate to start in a patient meaning that meaningful response is received in a more timely manner as a result. The background would be better	Thank you for your comment. I have included the following in the introduction (p2):<br><br>*The focus of most current germline pharmacogenomic tests has been in the context of reducing the likelihood of ADRs.[25] However pharmacogenomic information can*

written such that what PGx does and how it should be used is clearly stated and explained.	*also help improve drug effectiveness. For example, some drugs need to be activated by enzymes in the body, and genetic differences can affect how well this happens, such as CYP2D6 with codeine or CYP2C19 with clopidogrel; or genetic changes in drug targets can influence how well a drug works such as VKORC1 with warfarin or CFTR with ivacaftor.*
PGx is already delivered nationally through community pharmacies in the Nederlands and GP practices in Norway as part of their national health systems. I am unsure if community pharmacy is not included in the definition of 'primary care'. It is also available as a private service widely in primary care in Australia, Canada and USA. A GP based service is currently being trialled as an implementation study (The Progress Trial) in Manchester, UK, which includes GPs and Pharmacists. An economic model is being built by a health economist as a result,<br><br>I therefore think the background needs to be more clear as to where this project sits and what it will really add as is seems to suggest that the research will be performed on those naive to the use of PGx. It would be far more useful to perform in on those who have used it in primary care.	Thank you for your comment. I hope the reply below answers the reviewers questions.<br><br>We did approach the PROGRESS team at the outset of my project. We did also discuss the possibility of approaching participants from the PROGRESS study, however this required further ethical approval to enable us to approach those participants, which we decided was not feasible in with the timescale that I have.<br><br>We have approached PGX-naïve participants to understand their concerns about the potential use of PGx, as they will be the end-users of this technology.<br><br>The Personalised prescribing report published by the RCP/BPS, has highlighted the need for further research into the implementation of PGx in primary care.<br><br>This study is examining the challenges and benefits from UK HCP and patients' perspective. Also identifying which HCP could easily take on PGx testing and the cost effectiveness if led by that particular HCP. The PROGRESS study is a proof-of-concept study, with an economic analysis of a panel test as implemented according to the PROGRESS protocol. For this this study the economic analysis will be used to estimate the cost-effectiveness of different HCPs being involved in the delivery of a PGx service in primary care.<br><br>In terms of Primary care this includes GP services and community pharmacies, indeed community pharmacy is one of the arms in the implementation model for WS3 and this information is included in the introduction section (p2).
It is also very clear that most healthcare professionals have limited understanding of what PGx is and what it isn't. Therefore qualitative work to understand their perspectives is likely to not add a lot of value as views will be all theoretical.	Thank you for your comments:<br><br>Yes, I agree, but I feel it is important to understand the general feeling of the HCP and patients about the potential use of PGx testing in primary care.
I would have expected WP1 to be a review of the literature, as this would provide much better evidence and information for building an economic	Thank you for your comment:

model of this nature that qualitative views from intervention ignorant and naive individuals.	The main author (SQ) has published a SR in 2022, quoted in the introduction (reference 14) on the barriers and facilitators for implementation in primary care. From the SR it was clear that UK-based studies are lacking, my SR identified only one small study on GPs. My current study includes pharmacists, nurses and patients.<br>I intend to update the SR towards the end of my fellowship, as I identified only 5 further studies in Jan 2024, which did not change the findings of my SR. I know further studies have been published since I identified those 5 studies. As mentioned, my plan is to update the SR in 2026, which will also form part of background chapter of my thesis.
Different drugs have different quality of evidence for PGx and different actionable rates and different companies offer different panels. The NHS is starting to coalesce its thoughts as to what drugs it would like to include, with Clopidogrel already being planned. It may be easier and cleaner to focus on one drug with known outcomes and adverse events rather than panels which have different outcomes for each drug.	Thank you for your comment:<br><br>For WS2 and WS3 we discussed using examples of panels or individual drug-genes. We have decided to develop a case-scenarios for the focus group for clopidogrel and codeine as individual drugs and not use panel testing for the case scenarios. This decision was based on the fact that the single drug-gene test for clopidogrel has been introduced for the NHS and this example could easily be a real-life scenario. We choose codeine as the other example, firstly due to familiarity in primary care and also because of the MHRA guidance about codeine.
The authors talk about implementation and again this may differ for different therapies. Delaying the use of some treatments in anticipation of the PGx result may be appropriate in some instances but not others. It may be better to build models based on different drugs rather than panels and use this to inform which drugs should eventually be included in panels.	Thank you for your comment:<br><br>Our economic analysis is based on different models of service provision - based on the involvement of different HCPs along the care pathway. We agree that turn-around time will be more critical for some drugs compared with others. Our case studies will be based on specific medicines. The selection of genes for panels will not be fully aligned with urgency of result - CYP2C19 might be time critical for clopidogrel, for instance, but not for omeprazole.
Part of implementation is whether HCPs actually offer the test. The authors allude to this as being part of the problem but I am unsure if the level of implementation (reach) or quality of response to test results (dose/fidelity) will be considered in the model. PGx evidence comes from unstructured observational data and as such is of medium quality at best. Therefore, again, a good proportion of results will be incorrect for the patient, particularly if they are non-white. Again the authors do not seem to	Thank you for your comment:<br><br>We will be developing a full Health Economic Analysis Plan (HEAP) prior to the analysis. This will specify issues such as those highlighted by the reviewer, in detail. HCP compliance with ordering a test, when indicated, will be modelled explicitly. In PREPARE, for instance, "adoption of the DPWG recommendations was high, and overall, 69·9% of these recommendations were accepted by the physicians and pharmacists." The penetrance of testing and allele

recognise these issues in their plans.	frequency in relation with ethnicity, will be considered insofar as these are incorporated in existing economic analysis which will be used in our model of different service configurations.
The qualitative work requires review by a qualitative researcher. Either the authors are going to treat the data from the interviews as heterogeneous and therefore the planned numbers for some healthcare professional groups seem very small, or homogeneous and therefore a justification for different numbers for each group is required i.e. why should some voices get more say than others.	Thank you for your comment.<br><br>I have two very experienced qualitative researchers as part of my supervisory team (Prof Stephen Timmons and Dr Asam Latif) who have been instrumental in the advice and guidance throughout this project.<br>In terms of the sample, it is not meant to be statistically representative, and we are not treating the data from any group as either heterogenous or homogenous. The group sizes reflect the (broad) numbers likely to be involved, and also what is practical to recruit in the time and resources available. The number of participants per WS are<br><br>WS1 – GPs x6, pharmacists x6, nurses x6, patients x6<br>WS2 – HCP x16-24, patients x 8-12
The paper also needs reviewing by a health economist with expertise in modelling as I am unable to comment on this element.	
